# Expression of UGP2 and CFL1 expression levels in benign and malignant pancreatic lesions and their clinicopathological significance

**DOI:** 10.1186/s12957-018-1316-7

**Published:** 2018-01-18

**Authors:** Lingxiang Wang, Li Xiong, Zhengchun Wu, Xiongying Miao, Ziru Liu, Daiqiang Li, Qiong Zou, Zhulin Yang

**Affiliations:** 1 General Surgery Department, Second Xiangya Hospital, Central South University, Changsha, Hunan 410011 People’s Republic of China; 2 Research Laboratory of Hepatobiliary Diseases, Second Xiangya Hospital, Central South University, Changsha, Hunan 410011 People’s Republic of China; 3 Department of Pathology, Second Xiangya Hospital, Central South University, Changsha, Hunan 410011 People’s Republic of China; 4 Department of Pathology, Third Xiangya Hospital, Central South University, Changsha, Hunan 410013 People’s Republic of China

**Keywords:** Pancreatic ductal adenocarcinoma, Chronic pancreatitis, Pancreatic intraepithelial neoplasia, UGP2, CFL1

## Abstract

**Background:**

This study investigated UGP2 (uridine diphosphate-glucose pyrophosphorylase-2) and CFL1 (cofilin-1) expression in pancreatic ductal carcinoma (PDC), paracancerous tissue (PT), benign lesions (BL), and normal tissue (NT) and their clinicopathological significance.

**Methods:**

Surgical specimens, which were collected from 106 cases of pancreatic ductal carcinoma, 35 cases of paracancerous tissues, 55 cases of benign lesions and 13 cases of normal pancreatic tissues, were fixed with 4% formaldehyde to prepare conventional paraffin-embedded sections. EnVision immunohistochemical was used to stain for UGP2 and CFL1. Kaplan-Meier survival analysis was performed to assess the correlation of expression pattern with survival.

**Results:**

We found that positive UGP2 and CFL1 expression in PDC were significantly higher than those in PT, BL, and NT. In PT and BL with positive UGP2 and CFL1 expression, mild to severe atypical hyperplasia or intraepithelial neoplasia of grades II–III was observed in ductal epithelium. Positive UGP2 and CFL1 expression in cases with high differentiation, no lymph node metastasis, no surrounding invasion, and TNM (tumor-node-metastasis) staging I or/and II were significantly lower than those in cases with poor differentiation, lymph node metastasis, surrounding invasion, and TNM stage III and/or IV. Positive UGP2 expression in male patients was significantly lower than that in female patients. UGP2 and CFL1 expression in PDC were positively correlated. Kaplan-Meier survival analysis showed the degree of differentiation, tumor maximal diameter, TNM stage, lymph node metastasis, and surrounding invasion, and UGP2 and CFL1 expression were closely related to the average survival time of patients with PDC. The survival time of patients with positive UGP2 and CFL1 expression was significantly shorter than that of patients with negative expression. Cox multivariate analysis showed that poor differentiation, tumor maximal diameter ≥ 3 cm, TNM stage III or IV, lymph node metastasis, surrounding invasion, and positive UGP2 and CFL1 expression was negatively correlated with the postoperative survival rate and positively correlated with the mortality of patients with PDC.

**Conclusion:**

Positive expression of UGP2 and CFL1 can serve a valuable prognostic factor in pancreatic cancer.

## Background

Currently, pancreatic cancer is one of the most malignant tumors. Recently, pancreatic cancer has tended to occur at a younger age, and its incidence has exhibited a clear upward trend [1]. Pancreatic cancer has no obvious symptoms in the early stage, and thus, its early diagnosis is difficult. Because the pancreas has a rich supply of blood and lymph vessels and no capsule in the acinar, pancreatic cancer cells can easily invade the surrounding large blood vessels and nerves. Thus, regional lymph node metastasis and distant metastasis can occur even when the tumor is small. Therefore, most patients have reached the advanced stage by diagnosis and have missed their chance for radical surgical resection [2]. Identifying markers of pancreatic tumors for the screening of asymptomatic patients at the early stage has been a research hotspot in recent years. However, an ideal detection marker for the screening of pancreatic tumors at an early stage is not currently available [3]. Recent studies have found that the detection of mutations in the KRAS gene in bodily fluids, blood, and pancreatic juice may be a new auxiliary examination method for the diagnosis of pancreatic cancer; however, the clinical value of these mutations requires further verification [3]. Therefore, the exploration of new target molecules for the diagnosis and treatment of pancreatic cancer is particularly important.

Uridine diphosphate-glucose pyrophosphorylase-2 (UGP2) is an enzyme that functions in the glycogen biosynthesis process. Consisting of 508 amino acid residues with a relative molecular mass of approximately 56,000 Da, UGP2 catalyzes the reaction of uridine triphosphate (UTP) and glucose-1-P to generate UDP-glucose. A deficiency in this enzyme during the glycogen synthesis and decomposition processes leads to the clinical manifestations of glycogen storage disease. UGP2 is highly expressed in the skeletal muscle, followed by in the liver. The relationship of the UGP2 expression level with the occurrence and development of tumors has rarely been reported in the literature (only four studies, all of which are proteomics studies). These proteomics studies showed that the UGP2 expression level was significantly higher in cancer cells than in the corresponding normal cells or tissues [4–7].

CFL1 (cofilin, non-muscle isoform) is a high-affinity sphingosine-1-phosphate receptor. As an actin-binding protein, CFL1 plays an important role in cell proliferation and migration; functions in the occurrence, development, infiltration, and metastasis of tumors; and has received increasingly extensive attention. Based on the in-depth research of the CFL1 signaling pathway, blocking the EDG-1 signaling pathway may become an important strategy for the targeted therapy of malignant tumors. Recently, some studies have found that CFL1 is highly expressed in tumor tissues, including renal cell carcinoma [8], ovarian cancer [9, 10], oral squamous cell carcinoma [11], pancreatic cancer [12], breast cancer [13, 14], lung cancer [15], gastric cancer [16], and gallbladder cancer [17], and plays regulatory roles in triggering the transformation of tumor cells, enhancing the power of cells in transfer, and dividing cells. Studies have shown that inhibiting CFL1 activity in a malignant tumor may be a potential target for the inhibition of tumor progression and metastasis [9, 15].

This study applied the method of EnVision immunohistochemistry to investigate the expression levels of UGP2 and CFL1 in 106 cases of pancreatic ductal carcinoma, 35 cases of paracancerous tissues, 55 cases of benign lesions, and 13 cases of normal pancreatic tissues, as well as their clinical pathological significance. Furthermore, the relationship of the expression levels of UGP2 and CFL1 to the prognosis of patients with pancreatic ductal carcinoma was explored.

## Methods

### Clinical data

Surgical specimens from 106 cases of pancreatic ductal carcinoma with a pathological diagnosis of pancreatic ductal adenocarcinoma with no preoperative radiotherapy and/or chemotherapy were collected from Second Xiangya Hospital and Third Xiangya Hospital from January 2000 to December 2011. Among them, 61 cases were male (57.5%) and 45 cases were female (42.5%); 22 cases were aged ≤ 45 years (20.8%), and 84 cases were aged > 45 years (79.2%); the average age was 54.50 ± 11.53 years. The pathological types included 38 cases of highly differentiated adenocarcinoma (35.8%), 35 cases of moderately differentiated adenocarcinoma (33.0%), and 33 cases of poorly differentiated adenocarcinoma (31.1%). The maximum diameters of the mass were < 3 cm in 13 cases (12.2%), 3–5 cm in 68 cases (64.1%), and > 5 cm in 25 cases (23.5%). Extrapancreatic regional lymph node metastasis was pathologically diagnosed by biopsy in 29 cases (27.3%), and no lymph node metastasis was detected in 77 cases (72.6%). Invasion into the surrounding extrapancreatic tissues and organs was intraoperatively found in 64 cases (60.4%), and no invasion into the surrounding extrapancreatic tissues and organs was intraoperatively found in 42 cases (39.6%). The TNM (tumor-node-metastasis) clinical stage was Ι in 11 cases (10.4%), ΙΙ in 42 cases (39.6%), ΙΙΙ in 37 cases (34.9%), and ΙV in 16 cases (15.1%). Follow-up information was collected by mail or telephone interviews for the 106 patients with pancreatic ductal adenocarcinoma, with a follow-up period of 2 years. Three cases with a survival time > 2 years were included in the statistical analysis as censored cases. Among the 106 cases, a survival time of > 12 months occurred in 24 cases, with an average survival time of 9.57 ± 0.69 months. Additionally, 35 cases of paracancerous epithelial tissues adjacent to the above pancreatic ductal adenocarcinomas (≥ 2 cm from the cancer tissue) were collected, including 12 cases with a normal microscopic pathological diagnosis, 10 cases with mild atypical hyperplasia, 8 cases with moderate atypical hyperplasia, and 5 cases with severe atypical hyperplasia.

Surgical specimens of 55 cases with benign lesions were collected from the Department of Hepatobiliary and Pancreatic Surgery at the Second Xiangya Hospital from January 2000 to December 2011. Among them, 29 cases were male (52.7%), and 26 cases were female (47.3%). Thirteen cases were aged ≤ 45 years (23.6%), and 42 cases were aged > 45 years (76.4%). The pathological types included 20 cases of chronic pancreatitis (36.4%), 20 cases of adenoma (36.4%), and 15 cases of intraepithelial neoplasia (27.3%). The chronic pancreatitis was mild in 10 cases, moderate in 6 cases, and severe in 4 cases, including 3 cases of mild atypical hyperplasia in the glandular epithelium, 2 cases of moderate atypical hyperplasia, and 1 case of severe atypical hyperplasia. The adenomas included 15 cases of serous adenoma and 5 cases of mucinous adenoma, with mild atypical hyperplasia in the glandular epithelium in 4 cases, moderate atypical hyperplasia in 3 cases, and severe atypical hyperplasia in 2 cases. The intraepithelial neoplasia included 6 cases in grade I, 5 cases in grade II, and 4 cases in grade III.

As a control, surgical specimens from 13 cases of normal pancreatic tissues were collected from the Department of Hepatobiliary and Pancreatic Surgery at the Second Xiangya Hospital from January 2000 to December 2011. All of the normal pathological tissues were observed under a light microscope.

The above specimens were fixed with 4% formaldehyde for 24–48 h to prepare conventional paraffin-embedded sections with a slice thickness of 4 μm.

### Methods

Main reagents: Rabbit anti-human polyclonal UGP2 and CFL1 antibodies were purchased from Abgent Company (California, USA). The EnVision™ Detection Kit was purchased from Dako Laboratories (California, USA).

UGP2 and CFL1 were stained using the EnVision immunohistochemical method in strict accordance with the operation manual of the reagent. The main steps are as follows: slicing, dewaxing, and washing → 3% H_2_O_2_ in methanol for 10 min → trypsin for 15 min → dropwise addition of primary antibody at 37 °C for 60 min → dropwise addition of solution A at 37 °C for 30 min → development with a chromogenic solution for 15 min → light staining with hematoxylin for 1 min → dehydrating, clearing, and mounting with neutral balsam. Cells containing brown particles in the cytoplasm were considered UGP2 and CFL1 positive. The percentages of positive cells were determined by observing 400 cancer cells in 10 random fields in sections under high magnification. Cases with an average positive rate ≥ 25% were defined as positive cases, whereas cases with an average positive rate < 25% were defined as negative cases. The positive section provided by Beijing Zhong Shan Biological Technology Co., Ltd. served as a positive staining control, and replacement of the primary antibody with 5% fetal bovine serum served as a negative staining control.

### Statistical analysis

All of the experimental data were inputted into the SPSS 13.0 statistical package. The correlation of UGP2 and CFL1 expression with histological or clinical factors was analyzed using the *χ*^2^ test or Fisher’s exact test. Univariate survival analysis and the log-rank test were performed using the Kaplan-Meier method. A Cox proportional risk model was applied for multivariate analysis, the determination of the 95% confidence interval, and the normal approximation test (Wald’s test). *P* < 0.05 was considered significant.

## Results

### UGP2 and CFL1 expression and its significance

**Fig. 1:**
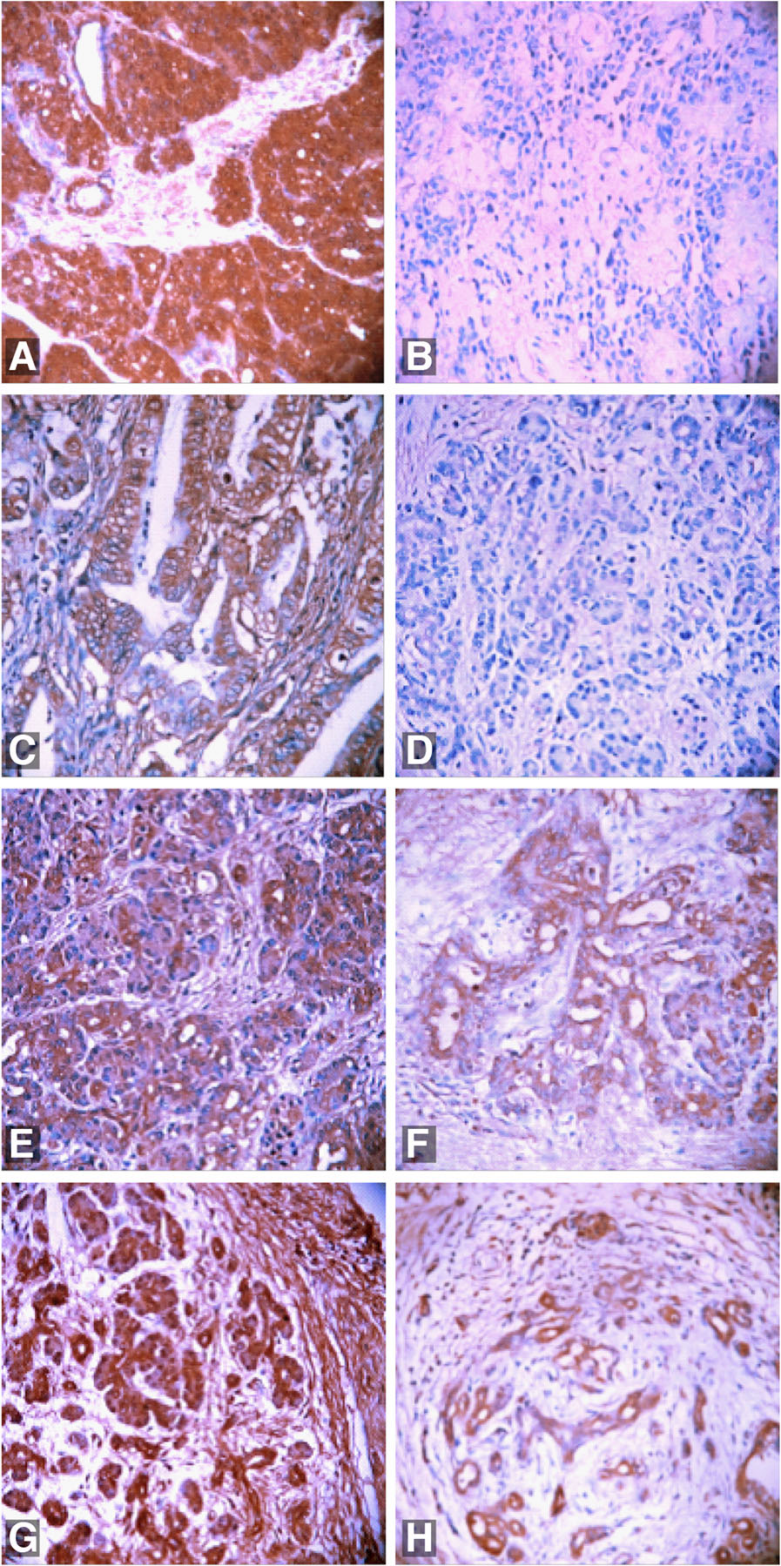
EnVision immunohistochemical staining (× 200) to evaluate positive (**a**) and negative (**b**) UGP2 expression in moderately differentiated adenocarcinoma, positive CFL1 expression in highly differentiated adenocarcinoma (**c**), negative CFL1 expression in moderately differentiated adenocarcinoma (**d**), positive UGP2 expression in paracancerous tissue (**e**), positive UGP2 expression in adenoma (**f**), positive CFL1 expression in grade II intraepithelial neoplasia (**g**), and positive CFL1 expression in chronic pancreatitis (**h**)

UGP2 and CFL1 were positively expressed in 62 (58.5%) and 56 (52.8%) of the 106 pancreatic ductal carcinoma cases, respectively. UGP2 and CFL1 were positively expressed in 10 (28.6%) and 9 (25.7%) of the 35 paracancerous tissue cases, respectively. UGP2 and CFL1 were positively expressed in 11 (20.0%) and 12 (21.8%) of the 55 benign lesions, respectively. Conversely, UGP2 and CFL1 expression was negative in all 13 normal pancreatic tissue samples. The positive expression rates of UGP2 and CFL1 were significantly higher in the pancreatic ductal carcinoma samples than those in the paracancerous tissues (*χ*^2^ = 9.426, *P* = 0.003 and *χ*^2^ = 7.786, *P* = 0.005, respectively), benign lesions (*χ*^2^ = 21.647, *P* = 0.000 and *χ*^2^ = 14.275, *P* = 0.000, respectively), and normal pancreatic tissues (*χ*^2^ = 15.874, *P* = 0.000 and *χ*^2^ = 12.973, *P* = 0.000, respectively). Additionally, the ductal epithelia of the paracancerous tissues and benign lesions with positive UGP2 and CFL1 expression showed mild to severe atypical hyperplasia or intraepithelial neoplasia (grades II–III) (Table 1). In the benign lesions, the positive expression rates of UGP2 in the samples with chronic pancreatitis, adenoma, and epithelial neoplasia were 15.0% (3/20), 25.0% (5/20), and 20.0% (3/15), respectively; the positive expression rates for CFL1 in the chronic pancreatitis, adenoma, and epithelial neoplasia samples were 20.0% (4/20), 20.0% (4/20), and 26.7% (4/15), respectively. Conversely, the positive expression rates for UGP2 and CFL1 did not significantly differ for the three types of benign lesions (*P* > 0.05; Fig. 1).

**Table 1:** The expression of UGP2 and CFL1 in PDC, PT, BL, as well as NT, and their significance

Type	Cases	UGP2 positive (%)	CFL1 positive (%)
PDC	106	62 (58.5)	56 (52.8)
PT	35	10 (28.6)**	9 (25.7)**
BL	55	11 (20.0)**	12 (21.8)**
NT	13	0 (0.0)**	0 (0.0)**

Compared with PDC: **P < 0.01

### The relationships between UGP2 and CFL1 expression and the clinicopathological characteristics of PDC

The positive expression rates of UGP2 and CFL1 in the cases with high differentiation, no lymph node metastasis, no invasion into the surrounding tissues and organs, and TNM stage I and/or II were significantly lower than the rates in the cases with poor differentiation, lymph node metastasis, invasion into the surrounding tissues and organs, and TNM stage III and/or IV (*P* < 0.05 or *P* < 0.01). Among all of the patients, 61 cases were male (57.5%), and 45 cases were female (42.5%). Additionally, 22 cases were aged ≤ 45 years (20.8%), and 84 cases were aged > 45 years (79.2%); the average age was 54.50 ± 11.53 years. The positive expression rate of UGP2 in male patients was significantly lower than the rate in female patients (*P* < 0.05). The age of the patients and the maximum diameter of the mass showed no significant relationship with the positive expression rates of UGP2 and CFL1 (*P* > 0.05). The data are shown in Table 2.

**Table 2:** The relationships between UGP2 and CFL1 expression and the clinicopathological characteristics of PDC

Clinicopathological characteristics	Cases	UGP2			CFL1		
		Positive (%)	*χ* ^2^	*P* value	Positive (%)	*χ* ^2^	*P* value
Age							
≤ 45 years old	22	13 (59.1)	0.004	0.949	14 (63.6)	1.301	0.254
> 45 years old	84	49 (58.3)			42 (50.0)		
Gender							
Male	61	30 (49.2)	5.130	0.024	31 (50.8)	0.233	0.629
Female	45	32 (71.1)			25 (55.6)		
Differentiation degree							
Highly differentiated	38	14 (36.8)	16.804	0.000	13 (34.2)	17.126	0.000
Moderately differentiated	35	20 (57.1)			16 (45.7)		
Poorly differentiated	33	28 (84.8)			27 (81.8)		
Maximum diameter							
≤ 3 cm	13	5 (38.5)	4.063	0.131	4 (30.8)	3.791	0.150
3–5 cm	68	39 (57.4)			36 (52.9)		
> 5 cm	25	18 (72.0)			16 (64.0)		
Lymph node metastasis							
No	77	38 (49.4)	9.684	0.002	33 (42.9)	11.233	0.001
Yes	29	24 (82.8)			23 (79.3)		
Invasion^a^							
No	42	13 (31.0)	21.728	0.000	15 (35.7)	8.178	0.004
Yes	64	49 (76.6)			41 (64.1)		
TNM stage							
I	11	3 (27.3)	20.074	0.000	2 (18.2)	11.171	0.011
II	42	17 (40.5)			20 (47.6)		
III	37	28 (75.5)			21 (56.8)		
IV	16	14 (87.5)			13 (81.3)		

^a^Invasion into the surrounding tissues and organs

### The relationship between the CFL1 and UGP2 protein expression levels in PDC

Thirty-nine of the 62 UGP2-positive cases were CFL1 positive, and 28 of the 44 UGP2-negative cases were CFL1 negative. There was a close positive correlation between CFL1 and UGP2 expression (*χ*^2^ = 7.261, *P* = 0.006).

### The relationships between the clinicopathological parameters and the UGP2 and CFL1 expression levels in PDC patients, with their average survival times

**Fig. 2:**
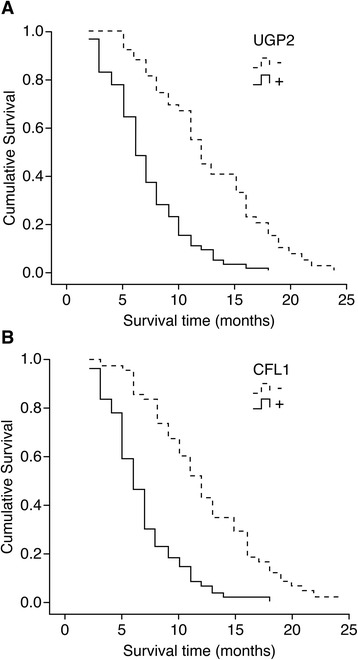
Survival curves of patients with positive and negative UGP2 expression in pancreatic ductal carcinoma (**a**) and positive and negative CFL1 expression in pancreatic ductal carcinoma (**b**)

Follow-up information was collected for the 106 patients with pancreatic ductal adenocarcinoma by mail or telephone interviews. The follow-up period was 2 years; patients with a survival time > 2 years were included in the statistical analysis as censored cases. Among the 106 cases, postoperative survival times ≥ 1 year occurred in 29 cases and < 1 year occurred in 77 cases, with an average survival time of 9.44 ± 0.69 months. The Kaplan-Meier survival analysis showed that the degree of differentiation, maximum tumor diameter, TNM stage, lymph node metastasis, and invasion into the surrounding tissues were closely related to the average survival times of the patients with pancreatic ductal carcinoma (*P* < 0.05 or *P* < 0.01). The survival times of the patients with positive UGP2 and CFL1 expression were significantly lower than the survival times of the patients with negative UGP2 and CFL1 expression (*P* = 0.000) (Table 3 and the survival curves in Fig. 2). The Cox multivariate analysis showed that poor differentiation, a maximum tumor diameter ≥ 5 cm, TNM stage III or IV, lymph node metastasis, and invasion into the surrounding tissues and organs were negatively correlated with the postoperative survival time and positively correlated with the mortality of the patients with pancreatic ductal carcinoma. All of the above variables were considered risk factors and independent prognostic factors. Positive UGP2 and CFL1 expression was negatively correlated with the postoperative survival time and positively correlated with the mortality of the patients with pancreatic ductal carcinoma. Thus, both of these proteins are risk factors and independent prognostic factors (Table 4).

**Table 3:** The relationships of the clinicopathological parameters as well as the expression of UGP2 and CFL1 in the PDC patients with their average survival time

Grouping	Number of cases (*N*)	Average survival (month)	Chi-square	*P* value
Sex				
Male	61	9.98 (2–24)	1.656	0.198
Female	45	8.61 (2–21)		
Age				
≤ 45 years old	22	8.18 (3–19)	2.144	0.143
> 45 years old	84	9.73 (2–24)		
Degree of differentiation				
Highly differentiated	38	11.27 (3–24)	17.786	0.000
Moderately differentiated	35	9.74 (3–21)		
Poorly differentiated	33	6.86 (2–14)		
Maximal diameter of the mass				
≤ 3 cm	13	13.46 (5–21)	7.504	0.023
3–5 cm	68	9.34 (2–22)		
> 5 cm	25	7.40 (3–24)		
TNM stage				
I	11	16.46 (11–24)	80.807	0.000
II	42	11.37 (3–22)		
III	37	7.14 (2–17)		
IV	16	4.56 (2–8)		
Lymph node metastasis				
No	77	10.64 (2–24)	27.120	0.000
Yes	29	6.35 (2–12)		
Invasion of the surrounding tissue				
No	42	13.33 (5–24)	46.949	0.000
Yes	64	6.83 (2–17)		
UGP2				
−	44	12.73 (5–24)	33.912	0.000
+	62	7.10 (2–18)		
CFL1				
−	50	12.28 (3–24)	36.767	0.000
+	56	6.77 (2–18)

**Table 4:** Cox multivariate analysis of the PDC patients’ survival rates

Grouping	Factor	B	SE	Wald	*P* value	RR	95% confidence interval	
							Lower	Upper
Degree of differentiation	Highly/moderately/poorly differentiated	.998	.385	6.720	.010	2.713	1.276	5.770
Maximal diameter of the mass	< 3 cm/3~5 cm/> 5 cm	1.536	.566	7.365	.007	4.646	1.532	14.089
TNM stage	I/II/III/IV	1.606	.588	7.460	.006	4.983	1.574	15.776
Lymph node metastasis	No/yes	1.983	.687	8.332	.004	7.265	1.890	27.925
Invasion of the surrounding tissue	No/yes	2.319	.789	8.639	.003	10.166	2.165	47.724
UGP2	−/+	1.833	.700	6.857	.009	6.253	1.586	24.656
CFL1	−/+	1.902	.695	7.489	.006	6.699	1.716	26.159

## Discussion

UGP2 deficiency in mammalian cells may lead to decreased glycoprotein synthesis and affect normal cell differentiation and integrity [18], indicating that UGP2 is essential for normal biological metabolism. UDP-glucose is the main activated form of glucose and serves as a glucosyl donor that is involved in the anabolism of sucrose, cellulose, hemicellulose, pectin, glycolipids, and glycoproteins [19]. The uptake of glucose in the human body can result in the formation of uridine diphosphate-glucose (UDPG), which is catalyzed by glucokinase, phosphoglucomutase, and UGP2. Additionally, UGP2 can couple with adenosine-5′-diphosphoglucose pyrophosphorylase (AGPase) to form ADPG, indicating that UGP2 also plays a role in the synthesis of starch [19, 20]. Recently, experimental proteomics methods showed that the UGP2 expression levels were significantly higher in some malignant tumors or malignant cells than in normal tissue or cells [4–7]. Tan et al. proposed that the UGP2 expression level could be used as an important bio-indicator for the progression and recurrence of hepatocellular carcinoma, with a high UGP2 expression level indicating fast progression, easy relapse, and a poor prognosis [5]. Thorsen et al. and de Jonge et al. reported similar results in colorectal cancer [6] and leukemia [7], respectively. No study on the UGP2 expression level in other epithelial malignancies, including pancreatic cancer, has been reported in the literature.

Tumor invasion and metastasis is a complex, continuous, and multi-step process that is dependent on the interaction between tumor cells and host cells. CFL is an important member of the actin depolymerizing factor/cofilin (ADF/cofilin) family. As a type 1 member, CFL1 is an important factor involved in the regulation of tumor cell metastasis and invasion and is closely related to the pathological processes of tumors [21]. CFL1 can promote actin, which is required for the formation of dendrites in the nuclei of adenocarcinoma and allows the exposure of the newly growing end of the F-actin at the outer periphery of the cell. Thus, CFL1 not only serves as one of the key factors that promotes the cell transition from a stationary state to a moving state but also plays an important role in the movement of tumor cells [21, 22]. CFL1 expression affects the tumor cell migration induced by growth factors and increases the migration speed of the tumor cells [21, 22]. Studies have found that the metastasis and invasion of breast cancer cells are affected by CFL1-mediated activation of a signal transduction pathway; thus, inhibiting the cofilin-mediated signal transduction pathway may be an effective method for breast cancer treatment [23]. A study of patients with lung cancer showed that CFL1 was highly expressed in lung cancer and was related to the invasiveness and metastasis of lung cancer cells; CFL1 expression in lung cancer cells is closely related to the prognosis and survival rates of the patients [24], suggesting that CFL1 can serve as a potential biological indicator to determine the lung cancer prognosis. This possibility has provided new insights for the diagnosis and treatment of lung cancer. In colon cancer cells, CFL1 is mainly expressed on the cell edge, which is also the expression site of F-actin, indicating that CFL1 can cause stronger metastatic potential of tumor cells. The decrease or increase in CFL1 pathway activity is mediated by LIM kinase-1 and results in the reduction and enhancement of metastasis, mobility, and vascular invasion of the corresponding tumor cells [25]. By activating actin aggregation, tumor cells can respond to chemotactic signals with the formation of pseudopodia in the targeted direction to extend the chain of actin and generate a new barbed end [26], which enables the determination of a precise direction of movement for the tumor cells. Therefore, CFL1 plays an important role in several aspects in tumor cells, including determining the migration speed, invasiveness, direction of motion, and nuclear division, and also affects the adhesion of the tumor cells and matrix.

The results of this study showed that the positive expression rates of the UGP2 and CFL1 proteins were significantly higher in pancreatic ductal carcinoma than those in paracancerous tissues and benign lesions (*P* < 0.05 or *P* < 0.01). In paracancerous tissues and benign lesions with positive UGP2 and CFL1 expression, mild to severe atypical hyperplasia or grade II–III intraepithelial neoplasia was observed in the ductal epithelium. The positive expression rates of UGP2 and CFL1 were significantly lower in cases with high differentiation, no lymph node metastasis, no invasion into the surrounding tissues and organs, and TNM stage I and/or II than in cases with poor differentiation, lymph node metastasis, invasion into the surrounding tissues and organs, and TNM stage III and/or IV (*P* < 0.05 or *P* < 0.01). The UGP2 and CFL1 expression levels were positively correlated in pancreatic ductal carcinoma (*P* = 0.006). The Kaplan-Meier survival analysis showed that the degree of differentiation, maximum tumor diameter, TNM stage, lymph node metastasis, and invasion into the surrounding tissues were closely related to the average survival of patients with pancreatic ductal carcinoma (*P* < 0.05 or *P* < 0.01). The survival time of the patients with positive UGP2 and CFL1 expression was significantly shorter than the survival times of patients with negative expression (*P* = 0.000). The Cox multivariate analysis showed that poor differentiation, a maximum tumor diameter ≥ 3 cm, TNM stages III or IV, lymph node metastasis, and invasion into the surrounding tissues and organs were negatively correlated with the postoperative survival time and positively correlated with the mortality of patients with pancreatic ductal carcinoma; these variables represent risk factors and independent prognostic factors. Positive UGP2 and CFL1 expression was negatively correlated with the postoperative survival time and positively correlated with the mortality of the patients; these variables represent risk factors and independent prognostic factors. These results demonstrated that the UGP2 and CFL1 expression levels reflected the progression, biological behavior, and prognosis of pancreatic ductal carcinoma. These proteins may play a coordinating role in the development of pancreatic ductal carcinoma, although the functional mechanism remains unclear and requires further investigation.

## Conclusions

In this study, expression levels of UGP2 and CFL1 were closely associated with tumorigenesis and progression of pancreatic malignancy. Positive expression of UGP2 and CFL1 can serve a valuable prognostic factor in pancreatic cancer.

## Abbreviations

BL: Benign lesions
CFL1: Cofilin-1
NT: Normal tissue
PDC: Pancreatic ductal carcinoma
PT: Paracancerous tissue
TNM: Tumor-node-metastasis
UGP2: Uridine diphosphate-glucose pyrophosphorylase-2
